# The Effects of Mucopolysaccharide Polysulphate on Hydration and Elasticity of Human Skin

**DOI:** 10.1155/2011/807906

**Published:** 2011-06-30

**Authors:** Rungsima Wanitphakdeedecha, Sasima Eimpunth, Woraphong Manuskiatti

**Affiliations:** Department of Dermatology, Faculty of Medicine Siriraj Hospital, Mahidol University, Bangkok 10700, Thailand

## Abstract

*Background*. Mucopolysaccharide polysulphate (MPS) has been used in medicine as an anti-inflammatory and antithrombotic agent for over 50 years. Its chemical structure permits considerable hydrogen bonding with adjacent water molecules, which effectively leads to hydration of the surrounding tissue. In addition, it stimulates endogenous hyaluronate synthesis, resulting in an increase in water-binding capacity and viscoelasticity of the skin. *Objective*. To study the efficacy of 0.1% MPS on hydration and elasticity of human skin. *Methods*. The first part of this study was a randomized double blind placebo-controlled study which included 60 female volunteers aged 30–45 years with dry skin, defined by Corneometer CM 825. The volunteers were treated with either 0.1% MPS or vehicle control. All subjects were asked to apply 1 g of cream to their face twice daily for a total period of 4 weeks. Skin hydration and elasticity were measured at baseline and week 4 with Corneometer CM 825 and cutometer MPA 580, respectively, at forehead and both cheeks. The second part of this study focused on the efficacy of 0.1% MPS on skin hydration after single application. 20 female volunteers aged 30–45 years with dry skin, defined by Corneometer CM 825, were recruited to the study. All subjects were asked to apply 2 g of 0.1% MPS cream on entirely randomly selected forearm. Skin hydration at the middle of both forearms was measured at baseline, immediately after application, and every 1 hour after application for a period of 10 hours. *Results*. 57 subjects (28 in vehicle control group, 29 in MPS) completed treatment protocol. The baseline skin hydration of both groups was not significantly different (*P* = 0.47). Hower, there was a statistically significant difference in skin hydration at 4 weeks between MPS and placebo group (*P* = 0.01). Skin elasticity was significantly improved at week 4 in both groups (vehicle-control, *P* < 0.01, and MPS, *P* < 0.01). However, no significant difference in skin elasticity between MPS and vehicle-control group was noted (*P* = 0.15). Lastly, there was a statistically significant improvement in skin hydration after a single application (*P* < 0.01). This improvement was maintained for 10 hours. *Conclusions*. MPS provided improvement of skin hydration but not skin elasticity in woman with dry skin, compared with vehicle control. And MPS improved the skin hydration for at least 10 hours after single application.

## 1. Introduction

Characteristics of cutaneous aging include atrophy of the dermis due to loss of collagen, degeneration of the elastic fiber network, and loss of hydration of epidermis [1]. The severity of dry skin increases with aging. Additionally, dry skin or xerosis is associated with age-independent molecular changes including epidermal expression of basal and differentiation-related keratins as well as premature expression of the cornified envelope protein, involucrin [2].

Mucopolysaccharide polysulphate (MPS), a naturally occurring organoheparinoid compound, has been used in medicine for over 50 years as an anti-inflammatory and antithrombotic agent for the treatment of numerous conditions, including osteoarthritis, thrombophlebitis, and for thromboembolism prophylaxis [3–11]. Skin permeability of MPS has been demonstrated by the achievement of therapeutic systemic effects, including prevention and treatment of local symptoms associated with peripheral vascular disorders, following topical administration [4].

Structurally speaking, MPSs are comprised of long unbranched polysaccharides with repeating disaccharide units. The chemical structure permits considerable hydrogen bonding with adjacent water molecules, which effectively leads to hydration of the surrounding tissue through its water retention property [12–14]. In addition, MPS has been shown to increase hydration by stimulating endogenous hyaluronate synthesis, leading to increments in water-binding capacity and viscoelasticity of the skin [14]. MPS can decrease premature aging by inhibiting skin-degrading enzymes, including elastase and hyaluronidase, and increase RNA levels of important extracellular matrix molecules, including glycosaminoglycans and proteoglycans [7].

The purpose of this study was to quantitatively assess the efficacy of 0.1% MPS on hydration and elasticity of human skin.

## 2. Material and Methods

We conducted a randomized double blind placebo-controlled study to determine if a measurable clinical effect on skin hydration and/or elasticity was achieved following the application of an 0.1% MPS-containing cream (Hirudoid mild cream, OLIC (Thailand), Ltd., Thailand). This study was divided into 2 parts. 

The first part was designed to study the efficacy of 0.1% MPS on hydration and elasticity of human skin. 60 Thai females between the ages of 30 and 45 were recruited for this part of the study. Participants were selected from individuals who responded to a posted announcement at the university hospital affiliated with this study. Respondents were selected to participate if they satisfied the following criteria: nondiseased facial skin, as confirmed the investigators; dry skin condition, defined by the Corneometer CM825 (Courage-Khazaka, Koln, Germany) reading of 60 or less; agreed to undergo a “washout period” of one month in which no topical medical treatment was allowed to apply to the face and no systemic treatment known to affect the skin could be taken; the following agreed to adhere to the entire study protocol. Exclusion criteria included: inability to provide informed consent; diseased facial skin normal skin hydration defined by the Corneometer CM825 reading of more than 60 inability to undergo the washout period or inability to adhere to the study protocol. Written informed consent was obtained from each participant.

All volunteers with confirmed dry skin (Corneometer value ≤ 60) were randomized to 2 separate study groups for application of either an 0.1% MPS-containing cream or an MPS-free cream base as a vehiclecontrol twice daily for four weeks. Skin hydration and elasticity were measured at baseline and week 4 with corneometer CM 825 and Cutometer MPA 580 (Courage-Khazaka, Koln, Germany), respectively, at forehead and both cheeks.

During the 4-week study period, the application of moisturizer, toner, foundation, concomitant medication, or topical treatment of any type was prohibited at the study drug treatment site. Only the use of the supplied cleanser (Cetaphil gentle skin cleanser, Galderma Laboratories, L.P., USA) was permitted. Sixty grams per volunteer of either MPS-containing or vehicle-control cream was given to volunteers. Vehicle-control cream was composed of mineral oil glycerin, emulgator, purified water, and liquid paraffin. These two types of topical treatments were in identical packaging, so that neither the volunteer nor the investigator knew which type of treatment was being administered. Volunteers were instructed to apply approximately 1 gram of the study cream all over their faces, avoiding the periorbital area, 2 times daily at morning and night after facial cleansing for 28 days. Volunteers were instructed to rinse eyes with tap water if any cream accidently entered the eyes.

Measurements of skin hydration and elasticity were obtained at baseline and then immediately after the 4-week period at forehead and both cheeks by two blinded investigators. The hydration value (obtained by the corneometer probe) was graded on a value in arbitrary units from 0 to 130 (for facial skin: 0–<50, very dry; 50–60, dry, and >60, sufficiently moistured). The elasticity value was obtained by using the Cutometer probe. The result was presented in 10 different values (R0–R9). The value of R5 reflects the elasticity of the skin. All of the measurements were measured in the temperature- and humidity-controlled room with temperature of 25°C and humidity of 60%.

The second part of this study was designed to study the efficacy of 0.1% MPS (Hirudoid mild cream, OLIC (Thailand), Ltd., Thailand) on skin hydration after single application. We conducted a randomized double blind placebo-controlled study by recruiting 20 female volunteers aged 30–45 years with dry skin, defined by corneometer CM 825 (Courage-Khazaka, Koln, Germany). All subjects were asked to apply 2 g of 0.1% MPS cream on entirely randomly selected volar forearm. Skin hydration at the middle of both volar forearms was measured at baseline, immediately after application, and every 1 hour after application for a period of 10 hours. All of the measurements were measured in the temperature- and humidity-controlled room with temperature of 25°C and humidity of 60%.

This study was approved by the Ethical Committee on Research Involving Human Subjects, Faculty of Medicine, Siriraj Hospital, Mahidol University, and conformed to the guidelines of the 1975 Declaration of Helsinki. Written informed consent was obtained from all study subjects.

### 2.1. Statistical Analysis

Unpaired *t-*test was employed to compare baseline values for hydration and elasticity between the study and control groups. Paired *t-*test was used to compare baseline hydration and elasticity to those obtained after the 4-week cream trial period. Repeated analysis of Variance (ANOVA) test was used to compare skin hydration between both volar forearms of each subjects at baseline, immediately after application, and every 1 hour after application for a period of 10 hours. Bonferroni posttest was also used to compare skin hydration between each of the time intervals. All statistical analyses were performed using SPSS software, version 16.0.

## 3. Results

For the first part of the study, fifty seven of the original sixty volunteers (29 MPS, 28 control; mean age 36 years in both groups) completed the 4 weeks of the study protocol. The average baseline values of skin hydration in MPS and vehicle-control group were 49.04 (±5.80) and 47.86 (±6.74) (*P* = 0.47). The average baseline value of skin elasticity (R5) in MPS and vehicle-control group were 54.24 (±8.28) and 54.95 (±9.26)(*P* = 0.76). 

**Figure 1:**
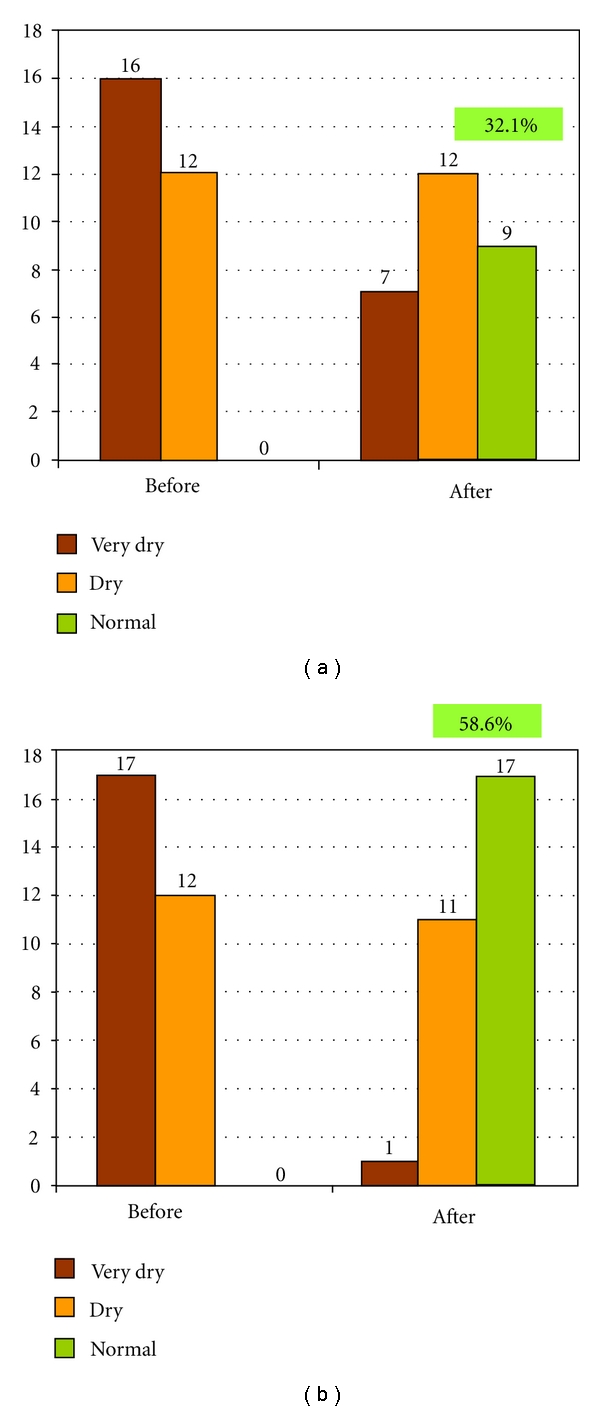
32.1% of vehicle-control subjects (a) and 58.6% of MPS subjects (b) demonstrated improvement to normal skin hydration after 4 weeks of application (*P* < 0.01).

Statistical analysis of skin hydration values as measured by the corneometer at baseline and after the 4-week study period a statistically significant improvement in hydration among both groups (Figures 1(a) and 1(b)). Additionally, a statistically significant difference in the degree of improvement in hydration was found when comparing the amount of increase in hydration between the control and study groups (*P* = 0.01).

**Figure 2:**
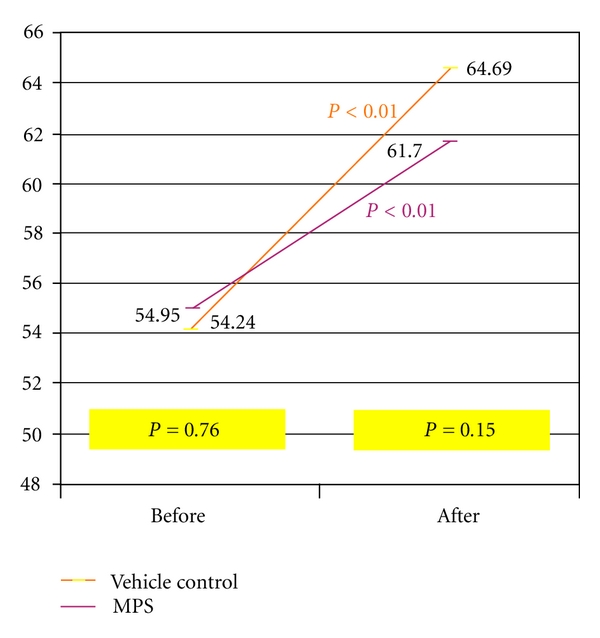
No significant difference in skin elasticity between both groups (*P* = 0.15).

Although the Cutometer at reading demonstrated a statistically significant improvement in elasticity among both groups after the 4-week period (Figure 2), no statistically significant difference in the degree of improvement in elasticity was found between the control and study groups (*P* = 0.15).

None of the volunteers reported any adverse reactions to either the control or study treatment throughout the study period. 

**Figure 3:**
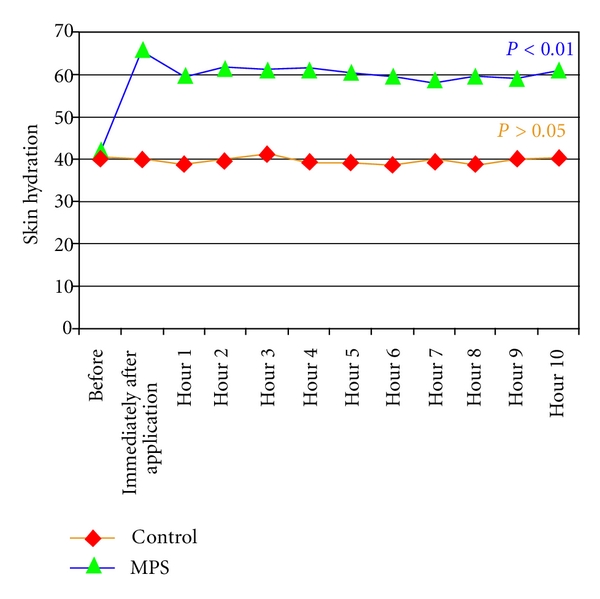
Skin hydration at MPS side was maintained for 10 hours after single application.

For the second part of the study, all 20 subjects completed the 10 hours of the study protocol. Statistical analysis verified that patient age, room temperature, and air humidity did not differ significantly during 10 hours of study period. The mean age of subjects were 33.0 years (SD = 2.71). The room temperature and the air humidity were set at 24.33°C (SD = 0.42) and 60.82%  (SD = 1.97), respectively. The average baseline values of skin hydration in MPS and control side were 41.87 (SD = 6.71) and 40.68 (SD = 7.67), respectively. Statistical analysis of skin hydration values as measured by the corneometer demonstrated that there was no significantly differences in skin hydration between the MPS and control side before treatment (*P* = 0.61). However, skin hydration of the MPS side improved significantly immediately after application, and this improvement was maintained for 10 hours after single application (*P* < 0.01) (Figure 3). None of the volunteers reported any adverse reactions to MPS throughout the study period.

## 4. Discussion

Mucopolysaccharide polysulphate in MPS cream is similar to the body's own mucopolysaccharides. The pharmacological properties of topically applied MPS correlate well with the documented clinical effects [15]. In the first part of the study, we demonstrated that MPS application twice daily over a 4-week period resulted in a significant improvement in skin hydration, compared to similar application of control cream base. Our findings may be explained by taking into consideration the chemical structure of MPS, a glycosaminoglycans (GAGs) derivative, which has abundant hydroxyl groups, and readily form hydrogen bonds with adjacent water molecules. The association of water with MPS molecules prevents evaporation of water, resulting in decreased desiccation of the surrounding skin. 

The finding that consistent MPS application improves skin hydration is an important finding as diminished skin hydration is associated with numerous skin conditions, including cutaneous photoaging and xerosis. Cutaneous photoaging correlates with focal deposition of GAGs within the mid- and deep dermis as compared to the diffuse distribution of GAGs found in healthy sun-protected dermis, in which these macromolecules are found through the dermis between collagen bundles [7]. Although sun-damaged skin contains increased amounts of glycosaminoglycans, given alterations in elastotic materials, these molecules may not be able to function in the typical mechanism as that of sun-protected skin. As a type of glycosaminoglycan, topical MPS application may provide a more even distribution of GAGs, similar to that of healthy non-UV-damaged dermis. These evenly distributed GAGs may then serve to promote hydration and aid in the movement of nutrients and cellular metabolites [16].

Xerosis, characterized clinically by pruritic, dry, cracked, and fissured skin with scaling, is increasingly common among the elderly because aged individuals have decreased sebaceous and sweat gland activity [16]. The cracks and fissures that characterize this condition result from epidermal water loss, and therefore this condition may be improved with the use of MPS.

Dermal absorption of MPS in human was investigated in vitro. The results demonstrated that MPS penetrated the skin and was able to reach dermal layers in effective concentrations [17]. However, the absorption of MPS to the systemic circulation was too low to influence blood coagulation [18, 19]. This could be interpreted that MPS may be “trapped” in dermis leading to increased epidermal water retention. Since MPS includes organoheparinoid compound, hyaluronic acid, glycosaminoglycans, and so forth. Hydration of the skin might be improved by the increasing of hyaluronic acid and glycosaminoglycans contents.

Notably, there was statistically significant improvement in elasticity among both groups when comparing baseline to after the 4-week period of application of either the cream base or the MPS-containing cream. However, MPS application did not result in significant improvement in skin elasticity compared to the control cream base, indicating that the physiologic affect of MPS on skin does not involve an increase in collagen production significant enough to result in a clinically detectable improvement in skin elasticity. Therefore, the other ingredients in vehicle cream base, for example, glycerin [20] and liquid paraffin [21], may be responsible for the improvement of skin elasticity in both groups.

In the second part of this study, we demonstrated that single MPS application resulted in a significant improvement in skin hydration for at least 10 consecutive hours, compared to similar application of control cream base. The results of this study indicated that MPS may serve as a beneficial therapy in the treatment of xerosis the finding that MPS affected may be explained by taking into consideration the chemical structure of MPS. MPS, a glycosaminoglycans (GAGs) derivative, has abundant hydroxyl groups, which readily form hydrogen bonds with adjacent water molecules [16]. The association of water with MPS molecules prevents evaporation of water, resulting in decreased desiccation of the surrounding skin.

## 5. Conclusions

In summary, this study demonstrated that MPS provided improvement of skin hydration but not skin elasticity in woman with dry skin, compared with vehicle control. MPS could also improve the hydration human skin for at least 10 hours after single application. Future study considerations should include evaluating the effect of MPS on specific xerotic skin conditions as well as determining optimal MPS concentrations for the treatment of such a condition.
